# Investing in women’s health, continuing the journey, one manuscript at a time

**DOI:** 10.4069/whn.2024.03.21

**Published:** 2024-03-29

**Authors:** Sue Kim

**Affiliations:** Mo-Im Kim Nursing Research Institute, College of Nursing, Yonsei University, Seoul, Korea

## The ongoing push to invest in women and women’s health

As readers are aware, March 8 is International Women’s Day and much attention is given to the Glass-Ceiling Index (GCI) at this time of year. The GCI focuses on the status of women in the workforce using 10 indicators analyzed for the 29 countries of the OECD (Organisation for Economic Co-operation and Development) [1]. Having celebrated its 10th anniversary in 2023 pointing to disparities across the relatively rich countries [2], the GCI revealed that progress for women continues to lag behind men in 2024, as well as over the past decade [1,3]. Iceland was ranked as the best place to be a working woman in 2024 and Nordic countries consistently maintained high ranks; whereas progress for women in South Korea, Japan, and Turkey has remained stagnant and at the bottom tier, i.e., women continue to face the biggest workplace obstacles. This gap in gender equality in working women and men is pointed out as being related to unequal access to education, workplace discrimination, and the ‘motherhood penalty’ of childcare burden [3].

In response to these challenges, the United Nations marked International Women’s Day 2024 with the theme “*Invest in women: Accelerate progress*” [4]. It is important to remember that investing in women does not simply imply changing our world to be more ‘woman-friendly,’ but to recognize that empowering women “can spark change and speed the transition towards a healthier, safer, and more equal world for all”, especially in response to our global ails of ecological crisis, conflict, and poverty [4].

## Continuing the publishing journey to promote women’s health

What then, can we do to align with this important mission of investing in and empowering women? As a scholarly journal, we believe our role is to help nurse researchers, educators, and clinicians be able to access and utilize studies that raise awareness of sex and gender-based analysis [5-7] and explore innovative ways to promote women’s health. Asians and Asian subgroups have been reported to be underrepresented in health research in North America [8], which undermines women’s health at the regional and global levels. As a journal located in South Korea but also seeking to serve the Southeast Asian and Western Pacific regions and beyond, we are particularly keen to share quality research on Asian women’s health issues. This is not only to highlight their needs but also to become a bridge that contributes unique perspectives and contextual knowledge to better address women’s health across the world.

With this aim in mind, the editorial team has made an important decision: the journal title has changed to *Women’s Health Nursing* (WHN), applied from Volume 30, Issue 1 (March 2024). WHN succeeds its former title *Korean Journal of Women Health Nursing* and is now the official journal of the Korean Society of Women Health Nursing. The website (https://e-whn.org/) maintains all former journal content and the journal continues to be a fully open-access journal. Its new submission site is https://submit.e-whn.org.

This title change to WHN intends to reflect our commitment to continuing scholarly excellence within Asia as well as emphasizing the focus to move beyond, further expanding to around the world. We are currently in the process of updating our information indexed in the following international databases: Scopus, MEDLINE, PubMed Central, ESCI (Emerging Sources Citation Index), and the DOAJ (Directory of Open Access Journals). Our hope is that our new title will convey a clear message to readers and researchers that WHN is committed to women’s health nursing at the global level.

In this line, we are more committed than ever to publishing high-quality manuscripts in English, which will widen research applicability and its impact. Also, as adhering to reporting guidelines (e.g., http://www.equator-network.org/home/, http://www.nlm.nih.gov/services/research_report_guide.html, etc.) enhances comprehensive description of the study, WHN will require authors to submit the appropriate checklist according to their study design. This will allow both reviewers and readers to better understand how the study was conducted, as well as discern the quality of the research and have greater trust in its implications. Potential authors are encouraged to review the strategies related to utilizing reporting guidelines [9].

We believe all of us have valuable opinions, experiences, and evidence to share as companions on this scholarly journey. I invite you to join us in investing in women’s health, one manuscript at a time.
